# Primary Pulmonary Meningioma With Associated Multiple Micronodules: A Case Report With Comprehensive Diagnostic Overview

**DOI:** 10.1002/cnr2.2123

**Published:** 2024-06-24

**Authors:** Daoqi Zhu, Zhuan Ou, Guangning Yan, Jiawang Cao, Enwu Xu

**Affiliations:** ^1^ Department of Thoracic Surgery General Hospital of Southern Theater Command, PLA Guangzhou China; ^2^ Department of Pathology General Hospital of Southern Theater Command, PLA Guangzhou China; ^3^ The First School of Clinical Medicine Southern Medical University Guangzhou China

**Keywords:** case report, lung neoplasm, primary pulmonary meningioma, surgical resection, VATS

## Abstract

**Background:**

Primary pulmonary meningioma (PPM) is an exceedingly rare neoplasm originating in the meninges within the lung. Despite sharing similarities with its central nervous system (CNS) counterparts, PPM presents unique diagnostic challenges and therapeutic considerations owing to its infrequent occurrence.

**Case:**

This case report describes a 73‐year‐old male who underwent chest computed tomography (CT), which revealed a mass in the posterior basal segment of the right lower lobe, suggestive of a low‐grade malignant tumor approximately 30–40 mm in size. Single‐port video‐assisted thoracoscopic surgery (VATS) was performed to resect the mass via localized lesion excision (lung wedge resection). Intraoperative frozen section pathology indicated a low‐grade malignant epithelial tumor, leading to a decision for maximal lung function preservation, considering the patient's advanced age. The surgical team opted for a localized excision to ensure negative margins. Histopathological analysis confirmed the diagnosis of epithelioid PPM, a rare subtype even among PPM cases (World Health Organization [WHO] Grade I). The patient was discharged 9 days after surgery without complications and resumed normal daily activities 1 month postoperatively. The rarity of PPM precludes a standardized treatment protocol, with surgical resection as the primary approach. However, the efficacy of adjunctive therapies remains uncertain due to limited evidence.

**Conclusion:**

This case report contributes to a better understanding of PPM and emphasizes the importance of a comprehensive diagnostic evaluation and individualized treatment planning for this rare entity.

## 1 Introduction

Primary pulmonary meningioma (PPM) is a rare type of neoplasm originating from the meninges within the lung [1]. Meningiomas are typically benign tumors arising from the meninges of the central nervous system (CNS), primarily affecting the brain and spinal cord [2].

The etiology of PPM proposed theories for the pathogenesis includes the migration of embryonic cells or the presence of ectopic tissues. Ongoing research is aimed at elucidating its precise pathogenesis [3]. Given its rarity and relative obscurity, PPM often poses significant diagnostic challenges, and its clinical attributes and optimal management remain the subjects of ongoing research and discourse. The diagnosis of PPM presents significant clinical and radiological challenges. This tumor often presents with features that mimic other conditions, and cytological analysis of aspiration biopsies may fail to yield definitive conclusions. On CT and PET/CT scans, PPMs may demonstrate characteristics akin to those of pulmonary carcinoid tumors, including variable radiotracer uptake. To establish a definitive diagnosis of PPM, it is imperative to exclude the possibility of metastatic dissemination from the CNS, which necessitates comprehensive neuroimaging of the cranium and spinal canal. The preferred management strategy for PPMs is surgical resection, which serves both diagnostic and therapeutic purposes. Nevertheless, the necessity and extent of such interventions remain debatable, particularly in asymptomatic patients. By reporting this case at the General Hospital of Southern Theater Command in June 2023, we aspire to enrich the knowledge base and enhance diagnostic precision and patient outcomes in the management of PPM. It is particularly important to differentiate the rarest epithelioid PPM from other pulmonary carcinomas, which also commonly occur in the peripheral areas of the lungs, present with clear, smooth boundaries on imaging, and have a higher malignancy, worse prognosis, and higher incidence rate.

## 2 Case

### 2.1 Investigations

A 73‐year‐old male with a history of chronic bronchitis and no long‐term medical treatment presented to a local hospital in June 2023 with a 1 week of fatigue without chills, fever, chest tightness, dyspnea, dizziness, headache, nausea, or vomiting. CT performed at a local hospital revealed a suspicious mass in the posterior basal segment of the right lower lobe, suggesting a low‐grade malignant tumor.

**FIGURE 1:**
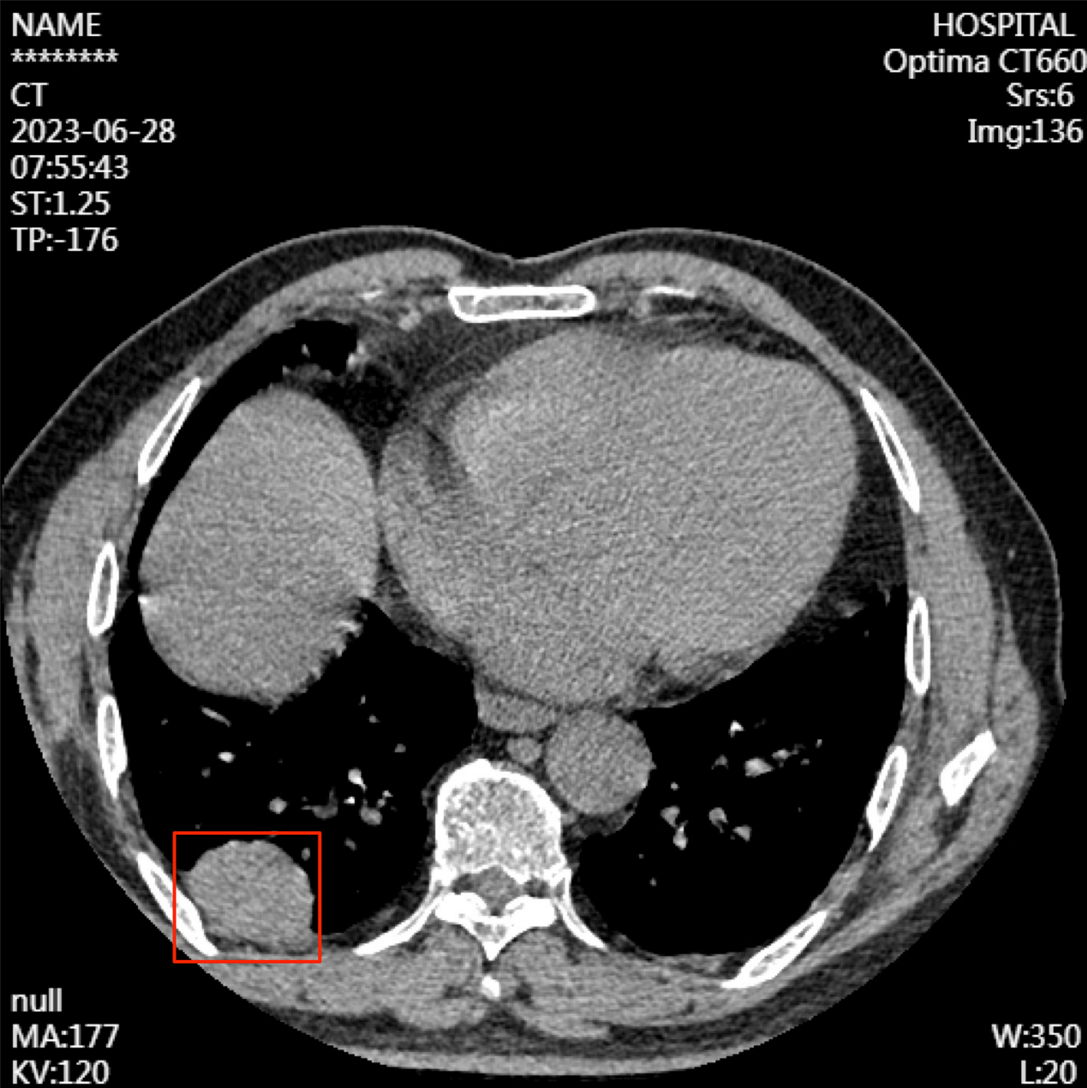
Chest‐enhanced computerized tomography disclosed an irregular soft tissue mass measuring approximately 41 mm × 29 mm × 34 mm. Labeled with red box.

Bilateral thoracic symmetry was observed with no apparent abnormalities in the bone structure. Pulmonary window images revealed normal transparency in both lung fields with coarse and disordered lung textures. An irregular soft‐tissue mass measuring approximately 41 × 29 × 34 mm was identified in the subpleural area of the posterior basal segment of the right lower lobe, displaying distinct margins and a relatively uniform density. The mass was broad and closely abutted the right posterior pleura, showing a pleural tail sign. Progressive enhancement was evident on contrast‐enhanced scans, with attenuation values measured at approximately 49, 70, and 74 Hounsfield units (HU) in the nonenhanced, arterial, and delayed phases, respectively. The pulmonary parenchyma adjacent to the right upper lobe was compressed (Figure 1).

**FIGURE 2:**
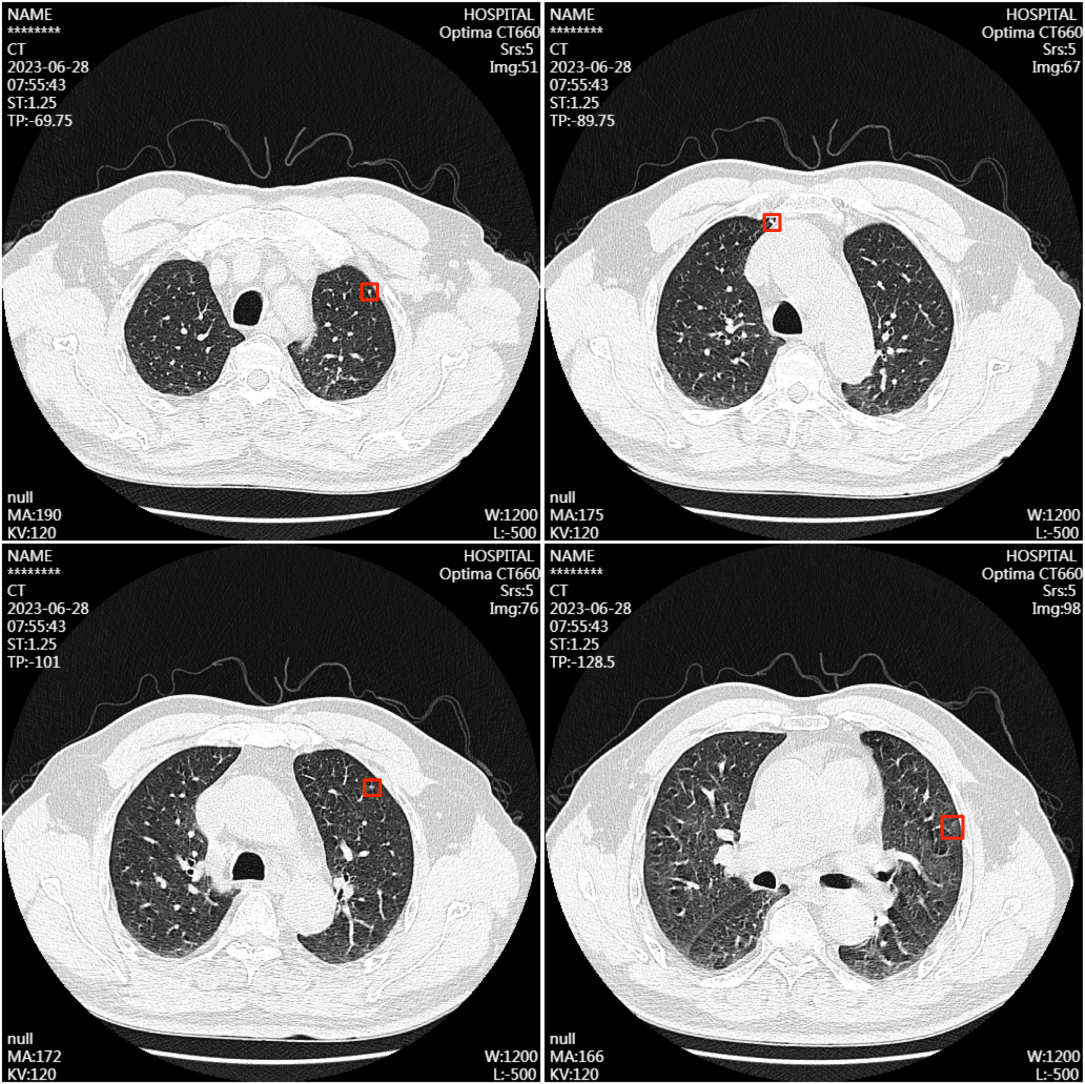
Chest‐enhanced computerized tomography disclosed several small nodular diameters of approximately 3–4 mm. Labeled with red box.

Thin‐section observations revealed several small nodular opacities in the apical and anterior segments of the left upper lobe, each in diameters of approximately 3–4 mm. Calcified nodular opacities are observed beneath the pleura in the anterior segment of the right upper lobe. Linear opacities of increased density were noted in the posterior segment of the left upper lobe and medial segment of the right middle lobe, displaying clear boundaries. No abnormal consolidations were detected in the remaining lung parenchyma and the bilateral hilae were not enlarged (Figure 2).

Mediastinal window images showed no deviation in the mediastinal structures, but an increase in cardiac volume was evident. The pulmonary artery widened, and no masses or enlarged lymph nodes were detected in the mediastinum. A thickened and adherent pleura was present on the right side, whereas the left pleura remained unaffected. No bilateral pleural effusion was identified. Bilateral thyroid nodules with slightly decreased density and clear margins were observed. A small cystic lesion with clear boundaries was observed in segment S4 of the liver tissue.

### 2.2 Admission and Treatment Course

The patient was then referred to our hospital for further evaluation and management. Comprehensive investigations and tests were performed to rule out contraindications to surgery. The patient underwent single‐port VATS for the resection of the right thoracic mass via localized lesion excision (lung wedge resection). During surgery, an intraoperative frozen‐section pathological examination indicated a low‐grade epithelial tumor. The wedge resection procedure was completed in approximately 20 min, and the entire surgical process proceeded smoothly without any complications. Postoperative care primarily focused on promoting coughing and expectoration as well as encouraging ambulation with morning activities. Given the low malignancy of the tumor, R0 resection was achieved. Considering the uncertain efficacy of adjuvant therapy in such cases, a decision was made to exclude additional treatment after surgery. Given that the additional nodules are less than 6 mm in size and their association with meningiomas remains uncertain, no specific intervention is warranted as per current guidelines. Consequently, these nodules were monitored during postoperative follow‐up.

### 2.3 Postoperative Pathological Findings and Diagnosis

**FIGURE 3:**
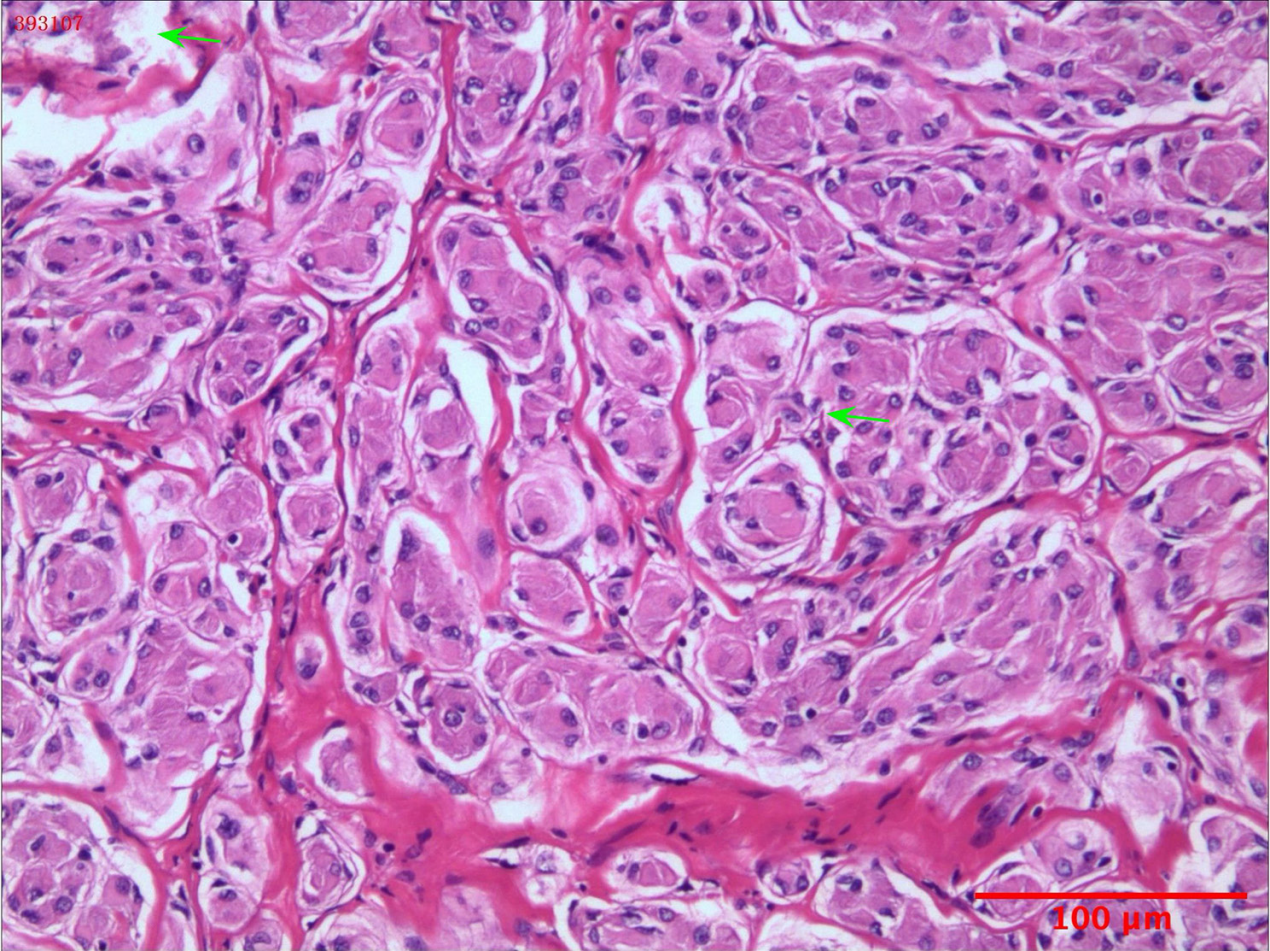
Histopathological results. Pathological analysis of surgical tissue. Nests of epithelioid tumor cells with abundant eosinophilic cytoplasm and mild nuclear atypia, the stroma was rich in collagen.

**FIGURE 4:**
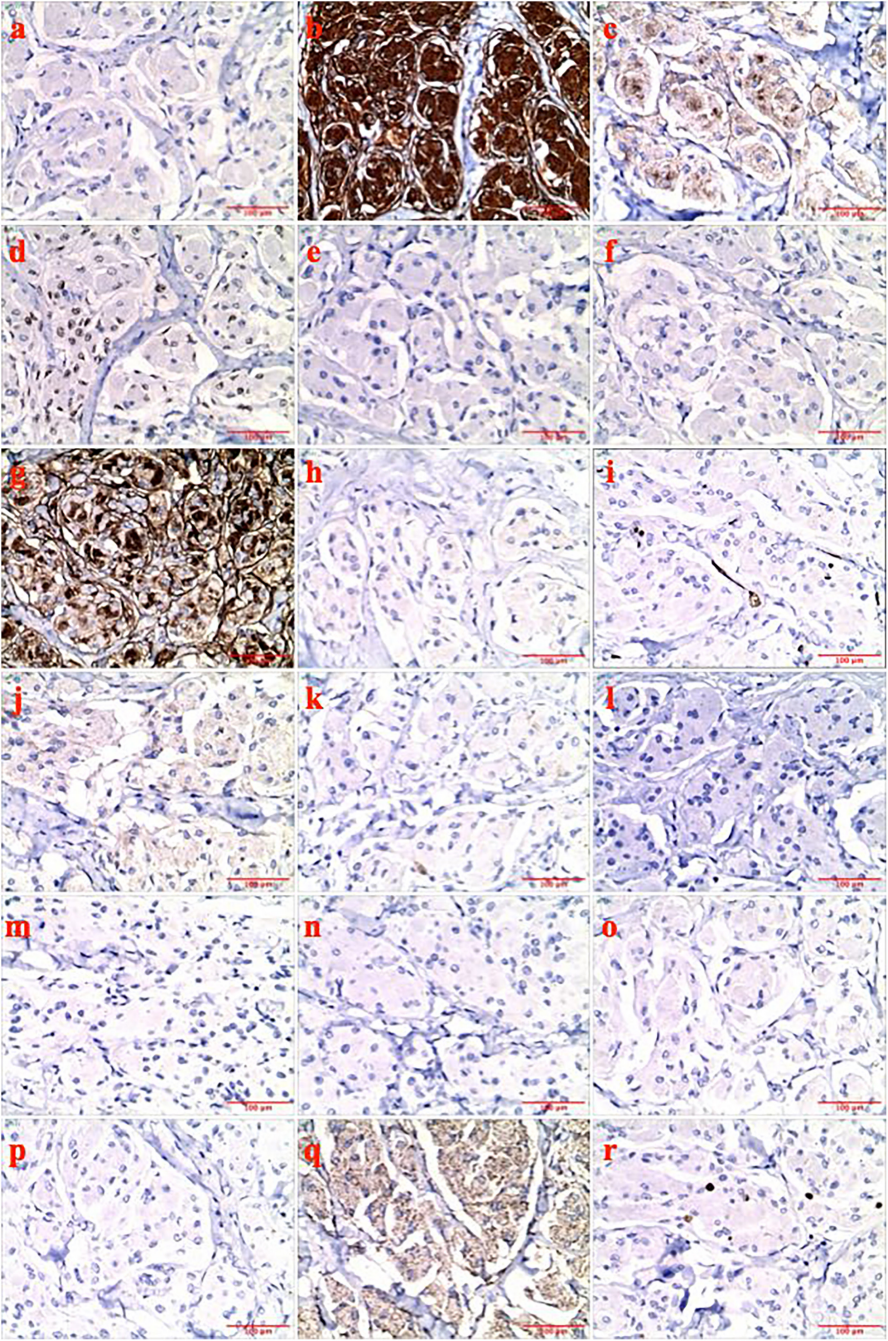
Immunohistochemistry results. (a) CK(−), (b) Vim(+), (c) EMA(+), (d) PR(+), (e) CgA(−), (f) Syn(−), (g) CD5/6(+), (h) Desmin(−), (i) CD34(−), (j) STAT6(−), (k) S‐100(−), (l) SOX‐10(−), (m) HMB45(−), (n) Melan‐A(−), (o) CR(−), (p) CK5/6(−), (q) WT1(cytoplasmic +), and (r) Ki67(+) ~3%.

Histopathological examination of the resected right lower lung mass revealed nests of epithelioid tumor cells with abundant eosinophilic cytoplasm and mild nuclear atypia. The stroma was rich in collagen and the tumor had clear margins with the surrounding lung tissue (Figure 3). Immunohistochemical analysis revealed staining of CK(−), Vim(+), EMA(+), PR(+), CgA(−), Syn(−), CD5/6(+), Desmin(−), CD34(−), STAT6(−), S‐100(−), SOX‐10(−), HMB45(−), Melan‐A(−), CR(−), CK5/6(−), and WT1(cytoplasmic +). The Ki67(+) index was approximately 3% (Figure 4a–r). The pathological diagnosis was confirmed as PPM, epithelioid‐type, World Health Organization Grade I, with a tumor volume of approximately 35 × 30 × 27 mm.

### 2.4 Postoperative Imaging

To ascertain whether the meningioma was primary or metastatic to the lungs, the patient underwent a postoperative cranial magnetic resonance imaging (MRI). Imaging revealed normal contrast differentiation between gray and white matter in both cerebral hemispheres. Notably, small hyperintense foci within the basal ganglia were observed on both T1‐ and T2‐weighted sequences, likely representing perivascular spaces. Additionally, FLAIR sequences revealed several regions with subtly altered signal intensities near the frontal lobes, vertex, and periventricular areas, which exhibited hyperintensity without post‐contrast enhancement. There was also slight ventriculomegaly and mild diffuse cerebral atrophy, as evidenced by the expansion of the sulci and subarachnoid spaces. The midline structures remain unshifted.

### 2.5 Follow‐Up

Postoperative care primarily focused on promoting coughing and expectoration as well as encouraging ambulation with morning activities. The patient was discharged 9 days after the surgery without complications and resumed normal daily activities 1 month postoperatively. At the 9‐month postoperative follow‐up, the patient exhibited favorable recovery following the surgical procedure. No adverse reactions or discomfort were reported during this period. However, the patient was reluctant to return to the hospital for subsequent reexamination.

## 3 Discussion

Ectopic meningiomas represent a fascinating subset of meningiomas that deviate from their usual intracranial location and arise at extracranial or non‐CNS sites. Although meningiomas are commonly associated with the cranial meninges, the occurrence of these tumors in ectopic locations presents unique diagnostic, pathological, and therapeutic challenges. Since the first reported case in 1982, approximately 70 cases have been described in the literature [4, 5, 6]. PPM has a distinct predilection for occurrence in elderly women, with this demographic group presenting a higher incidence [7].

Clinically, patients presenting with PPM may manifest nonspecific respiratory symptoms, including cough, hemoptysis, chest pain, or dyspnea [8]. In the realm of PPM, distinguishing imaging characteristics from those of other tumor types remains a significant diagnostic challenge. Chest CT scans commonly reveal solitary, well‐defined nodules or masses that may or may not exhibit calcification. Although some patients present with ground‐glass nodules or multiple cystic lesions, size alone cannot definitively differentiate between benign and malignant PPMs. Unfortunately, we could not distinguish any specific imaging characteristics in this case. Radiological evaluations commonly describe PPM, which are characterized by distinct boundaries and smooth contours. Consequently, differentiating PPM from pulmonary sarcomatoid carcinoma, which has a poor prognosis and high incidence rate, can be challenging when relying solely on imaging studies. Notably, maximum standardized uptake value (SUVmax) alone cannot reliably distinguish between benign and malignant PPMs. Even PPMs with mild fluorodeoxyglucose uptake can be benign, whereas those with low SUVmax values may be malignant [6]. Moreover, distinguishing PPM from other pulmonary neoplasms and metastatic meningiomas can be complicated and necessitates a comprehensive diagnostic approach.

Histologically, PPM resembles their CNS counterparts, characterized by the proliferation of neoplastic meningothelial cells forming distinctive whorls and psammoma bodies. The stroma was rich in collagen in this case compared with typical PPM cases. Immunohistochemistry plays a pivotal role in confirming the diagnosis, as these tumors typically express markers such as the epithelial membrane antigen (EMA, a sensitive and specific marker for meningiomas), vimentin (an intermediate filament protein expressed in mesenchymal and glial cells), and progesterone receptor (PR, a characteristic feature of meningiomas). Immunohistochemical findings from various studies indicate that in instances where relevant examinations were performed (excluding cases with insufficient examination details), a significant majority of patients were positive for vimentin and EMA. Conversely, a smaller yet notable subset was positive for S‐100 and keratin. Some PPM cases exhibit positive immunohistochemical markers such as Desmin and CD34. In the present case, all markers were negative. CD56 was positively expressed, whereas WT1 was expressed in the cytoplasm [4, 7, 9, 10]. Through meticulous documentation of immunohistochemical characteristics, it is possible to aggregate the molecular profile of PPM, with a particular focus on the uncommon epithelioid subtype, which is contingent on the availability of ample cases. This compilation of data provides critical clinical evidence that aids in distinguishing PPM from other pulmonary neoplasms, especially pulmonary sarcomatoid carcinomas, and suggests subsequent therapeutic strategies.

Given the rarity of PPM, a standardized treatment protocol has yet to be established. Surgical resection is commonly the primary therapeutic approach with the objective of complete tumor excision. However, the effectiveness of adjunctive therapies, including radiation or chemotherapy, remains uncertain due to limited clinical experience and scarcity of robust evidence. With the progress in high‐throughput sequencing and immunotherapy techniques, we propose the establishment of a diagnostic system for PPM based on liquid biopsy. This system identifies unique circulating tumor DNA molecules and facilitates early diagnosis and tailored treatment strategies. Given that most PPMs are benign tumors with favorable outcomes after surgical intervention, this approach may be particularly advantageous. For a subset of malignant PPMs, the development of a specific immunotherapy regimen informed by their immunomolecular characteristics could significantly improve patient management. This case report aimed to comprehensively and contemporaneously elucidate PPM, encompassing its clinical presentation, diagnostic intricacies, histopathological attributes, molecular features, and therapeutic strategies.

## 4 Conclusion

This case report comprehensively elucidates various aspects of PPM, including its clinical presentation, diagnostic intricacies, histopathological attributes, molecular features, and therapeutic strategies. Owing to the rarity of PPM, a standardized treatment protocol has not yet been established. Surgical resection is the primary therapeutic approach for complete tumor excision. However, the efficacy of adjunctive therapies remains uncertain owing to limited clinical experience and lack of robust evidence.

Radiological imaging, while essential, is insufficient to accurately distinguish PPM from other pulmonary tumors, underscoring the need for more definitive diagnostic modalities, particularly those that assess molecular characteristics. The management of benign PPM typically involves surgical resection. However, for infrequently malignant variants, a deeper understanding of their immunological profiles is imperative to devise individualized immunotherapy regimens. Considering the scarcity of epithelioid PPM, this report endeavored to meticulously document its molecular features, thereby contributing to the identification of early clinical diagnostic markers of PPM. Although the patient declined further examinations, leaving the status of other minor indeterminate nodules available, vigilant monitoring and heightened awareness will be sustained for this case and future PPM patients. This case study aimed to enrich the knowledge base and improve diagnostic precision and treatment outcomes of PPM. This case report contributes to a better understanding of PPM and provides valuable insights into its diagnosis and management.

## Author Contributions

**Daoqi Zhu:** conceptualization, writing–original draft, data curation. **Zhuan Ou:** methodology. **Guangning Yan:** methodology. **Jiawang Cao:** visualization. **Enwu Xu:** conceptualization, writing–review and editing, funding acquisition.

## Consent

This study obtained informed consent from the patient and approval from an ethics committee.

## Conflicts of Interest

The authors declare no conflicts of interest.

## Data Availability

The authors declare that the data supporting the findings of this study are available in the article. On reasonable request, a surgical video can be coordinated by contacting the corresponding author.
